# Association of DNA methylation with energy and fear-related behaviors in canines

**DOI:** 10.3389/fpsyg.2022.1025494

**Published:** 2022-12-14

**Authors:** Abigail R. Sanders, Neha Bhongir, Bridgett vonHoldt, Matteo Pellegrini

**Affiliations:** ^1^Department of Molecular, Cell, and Developmental Biology, University of California, Los Angeles, Los Angeles, CA, United States; ^2^Department of Ecology and Evolutionary Biology, Princeton University, Princeton, NJ, United States

**Keywords:** DNA methylation, canine (dog), epigenetics, behavior, genetics

## Abstract

**Introduction:**

Behavioral traits are influenced by gene by environment interactions. To study the genetic and epigenetic components of behavior, we analyzed whether dog behavioral traits could be predicted by their DNA methylation and genotypes.

**Methods:**

We conducted an analysis on dog behaviors such as sociability, trainability and energy as measured by Canine Behavioral and Research Assessment Questionnaire (C-BARQ) behavioral surveys paired with buccal swabs from 46 dogs. Previously we used targeted bisulfite sequencing to analyze DNA methylation and collected genotype data from over 1,500 single nucleotide polymorphisms (SNPs). Owner-reported C-BARQ responses were used to quantify 14 behavioral trait values.

**Results:**

Using Partial Least Squares (PLS) Regression analysis we found behavioral traits such as energy, attachment/attention-seeking, non-social fear, and stranger-directed fear to be significantly associated with DNA methylation across 3,059 loci. After we adjusted for age as a confounding variable, energy and stranger-directed fear remained significantly associated with methylation. We found that most behavioral traits were not predictable by our limited set of SNPs.

**Discussion:**

By identifying individual genes whose methylation is significantly associated with behavioral traits, we generate hypotheses about possible mechanisms involved in behavioral regulation. Overall, our study extends previous work in behavioral epigenetics, shows that canine behaviors are predictable by DNA methylation, and serves as a proof of concept for future studies in behavioral epigenetics.

## Introduction

Behavioral traits are influenced by gene by environment interactions. Genes influence an organism’s morphology and physiology, creating the innate framework for its learning, memory, and cognition (Breed and Sanchez, 2010). The environment interacts with an organism’s phenotype and can impact how it develops. Together, these genetic and environmental influences shape behavior (Breed and Sanchez, 2010). Gene by environment interactions have been studied across species to understand genetic influences on behavior. For example, Hunt et al. (2003) studied gene by environment interactions in honeybees to understand how animals can specialize within their communities to exhibit unique behaviors. They found that differences in honeybee guarding behaviors between 36 colonies were attributed to partial genetic dominance and environment interaction. Adaptive behaviors are also thought to be highly impacted by gene by environment interactions. For example, Madrid et al. (2018) found that the s-allele of 5-HTTLPR grants greater behavioral plasticity in rhesus macaques when combined with the environmental stimulus of high maternal protection. In addition, environmental and genetic influences impact aggressive behaviors. Sokolowski et al. (1997) found that in Drosophila certain alleles of cyclic guanosine monophosphate-dependent protein kinase, the *foraging* gene, influenced whether the fruit fly exhibited passive or aggressive foraging behavior. Wang et al. (2008) also noted that, in Drosophila, Cyp6a20 is a negative regulator of aggressiveness that is upregulated by high social interaction and this gene by environment interaction influences the level of aggressive behavior exhibited. Gene by environment interactions have also been explored in rodent models. One study in mice found that *Cntnap2* heterozygotes exposed to prenatal stress displayed altered sociability similar to *Cntnap2* knockouts, indicating a gene by environment interaction in neurodevelopment disorders (Papale et al., 2022). Another study examined genetic and environmental factors in impaired social and communication skills in mice, showing that the *Nlgn3/Cyfip1* pathway plays a role in shaping behavior and is influenced by the social environment (Sledziowska et al., 2020). Thus across species, gene by environment interactions have been shown to modulate varied behaviors.

The genetic influence on behavior stems from two factors: genotype and gene expression. Gene expression can be influenced by the environment, therefore it is an important factor in behavioral development. DNA methylation is frequently studied to analyze differences in gene expression. Champagne et al. (2006) noted that in rats, lack of maternal care by a parent is associated with increased methylation of estrogen receptors in the offspring and this methylation is associated with lack of maternal care by these offspring at reproductive age. Previous work in rodent models has also explored associations between DNA methylation and fear-related behaviors. Levenson et al. (2004) found that, in mice histone acetylation of histone H3 in the region CA1 of the hippocampus was regulated in fear conditioning and could be involved in long-term memory formation. Later, Bredy et al. (2007) found that histone deacetylation of brain-derived neurotrophic factor *(BDNF)* was associated with lessoning of conditioned fear response behavior in mice and Miller and Sweatt (2007) noted similar results in rat models, finding that contextual fear conditioning regulates DNA methylation levels in the hippocampus and upregulates expression of *de novo* methyltransferases DNMT3A and DNMT3B. Zovkic and Sweatt (2013) used DNMT activity findings to conclude that DNA methylation works with histone acetylation to regulate the storage of fear-based memory formations. Predator-induced fear has also been associated with changes in DNA methylation. Chertkow-Deutsher et al. (2010) found that higher *Dlgap2* methylation was associated with predator odor exposed rodents. St-Cyr and McGowan (2015) documented multigenerational DNA methylation associations, finding that the female offspring of mice exposed to predator odor during pregnancy have decreased methylation of *BDNF* in the hippocampus.

In the current study, we chose to focus on the domesticated dog to further our understanding of the relationship between epigenetics, genetics and behavior. Dogs are an excellent model system to examine behavioral epigenetics due to their unique population structure. The domestication of dogs is best understood under the lens of selective breeding and inbreeding. Dog breeding selects for specific physical and behavioral traits such as obedience for guide dogs, intelligence for herding, or trainability for police service. Artificial selection for desirable characteristics has often occurred through line breeding (inbreeding). In fact, one study analyzed 49,378 dogs across 227 different breeds and found a large inbreeding coefficient of *F*_adj_ = 0.249 (Bannasch et al., 2021). In extreme cases in which one male is used to continue a purebred line, inbreeding effective sizes have been as low as 50 individuals per breed (Calboli et al., 2008). Because inbreeding can simultaneously sustain a desirable purebred line, but also increase the likelihood of disease, dog breed pedigrees are often carefully maintained. Information regarding ancestry, relatedness, and inbreeding coefficients can provide insight on the selection of samples allowing researchers to better understand certain behaviors (Shearman and Wilton, 2011). In addition, dog breed demography has led to reduced genomic noise for trait mapping. Previous studies have found that dogs have large regions of the genome with linked alleles in strong linkage disequilibrium (Wall and Pritchard, 2003). These haplotype blocks have been found to be up to an order of magnitude larger than haplotype blocks found in humans (Gray et al., 2009). This enables the study of common social behaviors with fewer dogs and fewer genetic markers relative to human studies (Shearman and Wilton, 2011). The lack of genetic diversity in dogs makes them ideal candidates for behavioral mapping in a model organism.

Previous work has developed systematic methods to measure dog behaviors. One common approach to assess the behavioral characteristics of a dog is the Canine Behavioral and Research Assessment Questionnaire (C-BARQ) survey. This is a standardized tool for quantifying behavioral phenotypes in dogs and contains 101 behavioral questions which synthesize to quantify multiple behavioral axes (van den Berg et al., 2010). Survey details, tests of its reliability, and predictability have previously been described (Hsu and Serpell, 2003). Several studies have analyzed the predictability and reliability of the C-BARQ test for analyzing dog phenotypes. One such study used C-BARQ to characterize the behaviors of dogs relinquished to shelters (Duffy et al., 2014). Their analysis revealed that the C-BARQ assessment tool is a reliable and valid method for collecting behavioral data and screening dogs surrendered to shelters. Within the field, C-BARQ has proved to be an extremely useful tool used to quantify and study dog behavior.

Previous work has also examined the genetic basis of dog behaviors. Separation anxiety, touch sensitivity, owner-directed aggression, rivalry, and human-directed sociability have been found to have genetic components (Persson et al., 2015; Zapata et al., 2016). In a 2017 study researchers used the C-BARQ to investigate genetic characterizations of common dog traits in Labrador Retrievers (Ilska et al., 2017). The researchers studied single breed behavioral heritability and found “fetching” to have the highest heritability, *h*^2^ = 0.38, and six other traits to have heritabilities larger than 0.2 (Ilska et al., 2017). They also suggested that the traits they studied are polygenic and further research would require larger datasets to identify specific genes that influence behavior (Ilska et al., 2017). Previous research has also focused on interbreed behavioral heritability. One study analyzed 100,000 loci in the dog genome and compared 4,000 dogs with representation from 101 breeds (MacLean et al., 2019). They determined that the mean interbreed heritability for all 14 tested traits was 0.51, suggesting that the genome contributes a significant portion to behavioral variation across breed as well as within breeds. More recently, focus has shifted to understanding how environmental factors modulate the genome. The association between specific gene’s methylation and dog sociability has been studied previously, revealing that the methylation of the oxytocin receptor (*OXTR*) gene promoter was associated with dog social behavior (Cimarelli et al., 2017). While this finding is significant and demonstrates that gene regulation influences dog behavior, the focus was limited to a specific gene region. A broader analysis of associations between DNA methylation and social dog behaviors could uncover new information regarding how epigenetics can impact dog behaviors.

We hypothesized that a broad analysis of chromosome locations will reveal yet to be discovered associations between DNA methylation, genotype, and dog behaviors. Further, we sought to analyze the respective associations of DNA methylation and genotype on behavior to compare their relative influence on behavioral trait development. To address this hypothesis, we measured the association between behavioral phenotypes with genetic and epigenetic data. We collected and examined 14 behavioral traits including energy, attachment/attention-seeking, non-social fear, and stranger-directed fear in 46 dogs (Supplementary Table 1). We selected these 14 behaviors as they are defined by the C-BARQ questionnaire. We used the survey questions and behavior trait formulas provided by the C-BARQ to measure each behavioral trait. The DNA methylation and genotypes were obtained from a previous study that utilized targeted bisulfite sequencing (Rubbi et al., 2022). The targeted bisulfite sequencing methods utilized in Rubbi et al. (2022) are an advantageous approach for analyzing dog DNA methylation because bisulfite sequencing can be used on animals for which there is not an array and provides genotype information. The Rubbi et al. (2022) study produces several key findings that serve as the motivation for our current research. They found that DNA methylomes are significantly associated with dog age and can be used to create epigenetic clocks. In addition, they identify an association between methylomes and dog sex, body mass, and sterilization status. The study demonstrates that epigenetics can significantly predict physical characteristics. We aim to extend this analysis to the realm of non-physical characteristics, such as dog behaviors. Due to the small number of subjects involved and other limitations, our study can be considered a proof-of-concept study that begins to explore how future research can exploit the high power of dog genetics and epigenetics to understand the biology and environmental effects on behavior.

## Materials and methods

### Study design

Our work extended previous research in Rubbi et al. (2022), using their DNA samples to test associations between dogs’ DNA methylation, genotype, and behavioral traits. The 46 dogs included in our study were a subset of the 217 subjects analyzed by Rubbi et al. for which behavioral data could be collected. A request for behavior data was sent out to all owners, and collected for a subset of the previous subjects, and we cannot therefore exclude selection bias in our study design. For each of these 46 dogs, we administered behavior questionnaires to their owners to collect behavioral data. Then, our dog behavioral data were paired with the DNA methylation and genotype data collected in Rubbi et al. (2022).

In total we analyzed three datasets: methylation, genotype, and behavioral trait values. There were 46 dogs with complete epigenetic, genetic, and behavioral trait data. The dogs represented in the study came from 32 different dog owners. Dog ages ranged from 1 to 16 years with a median of 7 years. There were 31 unique breeds represented in the dataset and “*Australian Shepherd*” was the most common breed, representing 12 dogs. There were 27 female dogs in the study and 19 male dogs. All dogs had both genotype and methylation data as these were generated from the same buccal swab samples.

The dataset for behavioral trait values contained responses to the 42 questions in the C-BARQ questionnaire and calculated values for each of the 14 behavioral traits for each dog. The methylation dataset contained methylation values for 5,610 sites before filtering. After removing sites with missing data, we kept 3,059 sites. The genotype dataset contained 1,656 single nucleotide polymorphisms (SNPs) prior to filtering. After removing SNPs with missing data, the genotype data contained 930 loci.

### Quantitative behavioral traits

We collected behavioral data from 46 dog owners in the United States using the C-BARQ dog behavior questionnaire. We administered an abridged version of the traditional C-BARQ survey with 42 behavioral questions about common responses to stimuli as detailed in Supplementary File 1 (Duffy et al., 2014). The C-BARQ assesses each behavioral phenotype through situational questions. For example, “*How often does the dog bark persistently when alarmed or excited?*” and the owner responds on a scale of 1 to 4 (never to always). There are 14 standardized equations for calculating quantitative behavioral values based on individual question responses and these can be found in Supplementary File 2. For example, the “*excitability*” score is estimated as the average from responses to questions 1 and 2. Additionally, data were collected on breed, age, birth date, sex, weight, diet, and sterilization status (spayed/neutered). All sample collection procedures have been previously described (Rubbi et al., 2022).

### Targeted DNA methylation sequencing and single nucleotide polymorphism annotation

All DNA methylation and genotype data were obtained from Rubbi et al. (2022) where researchers generated dogs’ DNA methylation profiles, SNP genotyping, and SNP annotations. In that study, DNA from buccal swabs was extracted to generate DNA methylation profiles using targeted bisulfite sequencing (TBS-seq). Buccal study consisted of 70% epithelial cells and 30% immune cells (Rubbi et al., 2022). The complete bisulfite sequencing process utilized in Rubbi et al. (2022) is described in Supplementary File 3, Farrell et al. (2020), and Martin (2011). After bisulfite conversion, samples were aggregated into a methylation matrix (Morselli et al., 2021). Captured probes were selected to include loci whose methylation was associated with age (Thompson et al., 2017) or that were hyper conserved across mammals (Colwell et al., 2018). We overlapped the 5,608 probes used in our analysis with the CanFam4 genome and found 1,126 probes to be located on CpG islands (Kent et al., 2002). SNP annotation and genotyping procedures were also reported previously in Rubbi et al. (2022) and detailed in Supplementary File 3.

### Behavior clusters

We utilized the Python *Seaborn Clustermap* package to create a hierarchical clustered heatmap to show behavioral trait-breed correlations (Waskom, 2021). To analyze variation between dogs of the same breed and of different breeds, each dog was given a unique breed identifier such as “Australian Shepherd 4.” We also utilized Python *Seaborn Clustermap* package to create a second cluster map to show trait-trait correlations (Waskom, 2021). After creating a correlation matrix to capture correlation between traits, we used *Seaborn Clustermap* to group traits on both the *x* and *y*-axes.

### Predictability of behavior

We used Partial Least Squares (PLS) Regression from the Python *Cross Composition Module* in the *scikit-learn* package to analyze the relationship between each behavioral trait and the methylation/genotype data (Pedregosa et al., 2011). PLS is a widely used machine learning method used to determine a linear regression model by projecting both the predicted and observable variables to a new space.

Using PLS, we designated the methylation or genotype data as the predictors of behavioral phenotypes. Ultimately, we developed a supervised model for each behavioral trait that was trained using a Leave-One Out-Cross-Validation (LOOCV) method from the Python *scikit-learn* package (Pedregosa et al., 2011). For each trait, we aimed to avoid overfitting by creating a model with the highest predictive power that used a limited number of components. We created 12 models for each behavioral trait, varying the number of components, and therefore the number of predictor variables used. Components ranged from 1 to 12. We determined the optimal number of components by selecting the model with the highest correlation coefficient. The model with the optimal number of components was then utilized to calculate the correlation coefficient and *p*-value for that behavioral trait. We adjusted the *p*-values by applying a Benjamini Hochberg correction with the following formula:


$$P\,a\,d\,j=\text{p-value}*\frac{n\,u\,m\,T\,r\,a\,i\,t\,s}{r\,a\,n\,k}$$

Where *Padj* is the Benjamini Hochberg correction, *p*-value is the uncorrected *p*-value of the correlation coefficient, *numTraits* is the number of behavioral traits tested (14), and *rank* is the rank of the *p*-value (smallest to largest) out of all traits tested.

We used *pyplot* from Python’s Matplotlib and the top two components from the aforementioned PLS analysis to create a plot displaying how each component contributes to predicting a trait (Hunter, 2007). The *x*-axis represents the predictive power of component 1 while the *y*-axis represents the predictive power of component 2.

### Age as a predictor of behavior

We addressed possible behavioral age dependencies using two methods. In the first, we computed the association between age and behavioral traits. We used *corrcoef* from Python NumPy Library to compute the correlation between dog age and behavioral trait values (Harris et al., 2020). To evaluate age as a confound, we compared the r-squared values of age and the methylation model. If the methylation model’s r-squared value was greater, we determined that methylation contributed additional information for predicting dog behavior.

In the second approach, we regressed age out of the PLS methylation model. First, we used Ordinary Least Squares (OLS) Regression from Python *StatsModels* to find the behavioral trait values predicted by age (Seabold and Perktold, 2010). We generated four OLS models each with age as the predictor for one of the four methylation-predicted behavioral traits. Then, we calculated residual behavioral trait values by subtracting the observed behavioral trait values from the behavioral trait values predicted by the OLS age model. We used PLS regression to test if methylation values were predictive of the residual behavior values. The methods of the age-adjusted PLS models followed the methods for the unadjusted methylation models described in the previous section. For each behavioral trait, we calculated correlation coefficients, and *p*-values as summary statistics.

### Manhattan plots

We visualized results using Manhattan plots to display the relationship between a locus’ statistical significance in predicting behavior using DNA methylation as -log(*p*-value) in the *y*-axis against its position in the genome in the *x*-axis. The *p*-value was calculated using OLS Regression from *StatsModel* (Seabold and Perktold, 2010). Though age and methylation were modeled as predictors of behavior, only the *p*-value associated with methylation was plotted on the Manhattan plots. A Bonferroni threshold value was determined using a false discovery rate of 0.05 and sample size of 3057. Points with a -log(*p*-value) greater than the threshold value were labeled on all Manhattan plots. We adjusted the *p*-values by applying a Bonferroni correction with the following formula:


$$P\,a\,d\,j=\frac{p-v\,a\,l\,u\,e}{n\,u\,m\,T\,r\,a\,i\,t\,s}$$

Where *Padj* is the Bonferroni correction, *p*-value is the uncorrected *p*-value of the correlation coefficient, and *numTests* is the number of tests performed (3057).

### Statistical corrections

We used the Benjamini Hochberg Procedure to address the low power of the study and to correct for multiple testing. The derivation and justification for the Benjamini Hochberg Procedure to correct for multiple testing is described in Benjamini and Hochberg (1995). Correcting for multiple testing reduces the likelihood of Type 1 errors in our study. We used the Benjamini Hochberg equation explained in section “Predictability of behavior” to calculate the adjusted *p*-values and used 0.05 as the significance threshold.

## Results

### Quantitative behavioral traits

**FIGURE 1:**
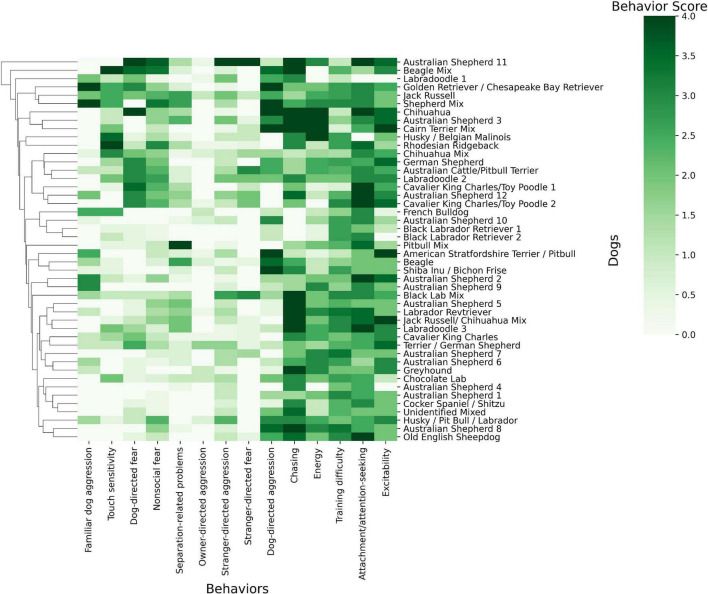
Heat map of the dog phenotype data and breed using Python *Seaborn Clustermap* (Waskom, 2021). The map depicts dog’s breeds and their scores from 0 to 4 in the 14 behavioral phenotypes. White colors indicate a score of 0 for a behavioral phenotype while darkening shades of green indicate a behavioral score close to 4. Related breeds and traits are clustered close to one another on nodes of the hierarchical trees. These nodes are determined by hierarchical clustering.

Behavioral trait values were computed across our cohort and compared against dogs’ breed (Figure 1). Though unexpected, Figure 1 revealed no clear clustering between dogs of the same breed regarding their behavioral phenotype scores. For example, there were 12 Australian Shepherds in our analysis and they did not cluster together and their scores (*r*_mean_ = 1.39, variance = 1.76) did not differ from that of the entire population (*r*_mean_ = 1.48, variance = 1.64). In the comparison of the 12 Australian Shepherds to all other breeds, no effect was observed.

**FIGURE 2:**
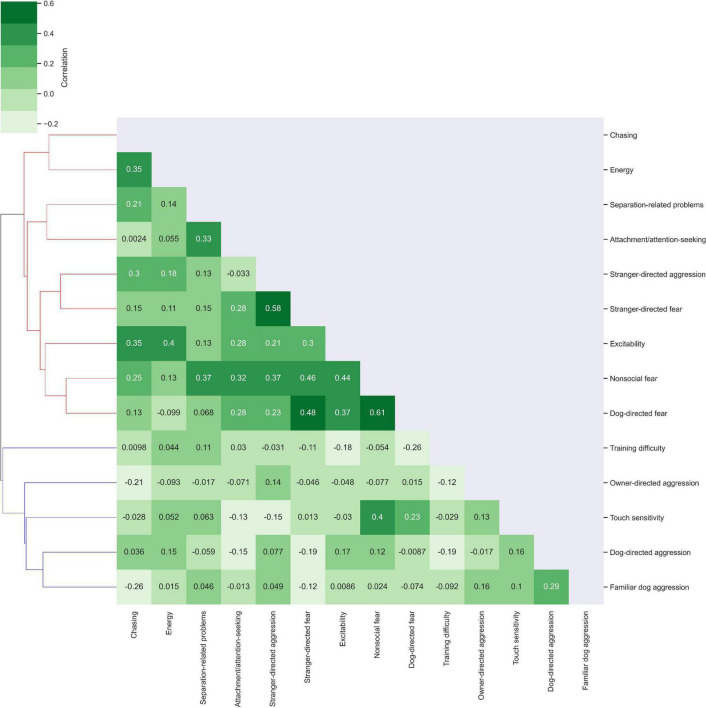
Cluster analysis of correlations between traits. Traits were examined for their correlation with other traits using Python *Seaborn Clustermap* (Waskom, 2021). Correlations of *r* = 1 are depicted on the diagonal as each trait correlates exactly with itself. Lighter green shades indicate low trait correlation while darker green shades indicate correlations closer to 1. Cluster 1 is colored in red on the dendrogram while Cluster 2 is colored in blue.

The trait correlation map in Figure 2 revealed two clusters of related traits. Cluster 1 consisted of excitability, stranger-directed aggression, stranger-directed fear, non-social fear, dog-directed fear, separation-related problems, attachment/attention-seeking, chasing, energy. Within Cluster 1, non-social fear and dog-directed fear were most correlated (*r* = 0.61). Stranger-directed fear and stranger-directed aggression were also highly correlated traits (*r* = 0.58). In comparison to the entire population (*r*_mean_ = 0.161, variance = 0.087) disregarding cluster assignment, Cluster 1 has a higher correlation and slightly lower variance (*r*_mean_ = 0.209, variance = 0.084). Cluster 2 was composed of training difficulty, owner directed-aggression, touch sensitivity, dog-directed aggression, and familiar dog aggression. Dog-directed aggression and familiar dog aggression were the most correlated traits within this cluster (*r* = 0.29). In comparison to the entire population (*r*_mean_ = 0.161, variance = 0.087), Cluster 2 had a much lower mean correlation coefficient and slightly smaller variance (*r*_mean_ = 0.074, variance = 0.083). Compared to the traits in Cluster 2, the traits in Cluster 1 were on average more correlated to one another.

### Predictability of behaviors

Partial Least Squares Regression analysis with LOOCV was used to construct models. Five models showed a significant correlation between the predicted and actual values of the traits (Table 1). PLS Regression with LOOCV was also used to construct models using the SNPs and one of these also yielded a significant correlation between the predicted and actual trait values. Correlations were determined to be significant if their *p*-value was below 0.05. Table 1 displays a summary of these results.

**TABLE 1:** Behavioral phenotype results.

Trait	Methylation					Genotype				
		
	Correlation coefficient (*R*-value)	*R* ^2^	*P*-value	p-adj	Components in model	Correlation coefficient (*R*-value)	*R* ^2^	*P*-value	p-adj	Components in model
Energy	0.4908	0.241	0.000534	0.00748	5	0.1065	0.0113	0.4812	0.674	1
Attachment/attention- seeking	0.4411	0.195	0.0022	0.0154	2	0.2289	0.0524	0.1261	0.441	1
Non-social fear	0.3767	0.142	0.0099	0.0462	6	0.1420	0.0202	0.3469	0.694	1
Stranger- directed fear	0.3736	0.140	0.0105	0.0368	5	−0.0025	6.25E-6	0.9895	0.990	1
Dog-directed fear	0.3150	0.0992	0.0330	0.0924	2	0.2504	0.0627	0.0933	0.435	1
Touch sensitivity	0.2856	0.0816	0.0544	0.127	3	0.4098	0.168	0.004691	0.0657	2
Chasing	0.2821	0.0796	0.0575	0.115	4	0.0447	0.002	0.7680	0.827	3
Dog-directed aggression	0.2523	0.0637	0.0907	0.159	1	0.1839	0.0338	0.2212	0.516	1
Familiar dog aggression	0.2181	0.0476	0.1456	0.226	1	0.2664	0.0710	0.0736	0.515	2
Separation- related problems	0.1632	0.0266	0.2785	0.390	2	−0.1164	0.0135	0.4427	0.774	10
Training difficulty	−0.1269	0.0161	0.4041	0.514	1	−0.2131	0.0454	0.1552	0.435	6
Owner-directed aggression	0.0818	0.00669	0.5889	0.687	3	−0.0476	0.00227	0.7564	0.882	1
Excitability	0.0506	0.00256	0.7384	0.795	1	0.0998	0.00996	0.5093	0.648	3
Stranger- directed aggression	0.0488	0.00238	0.7474	0.7474	5	−0.1155	0.0133	0.4466	0.695	1

Summary of the statistics of the PLS regression models of methylation data and dog behavior as well as genotype data and dog behavior. The table details correlation between data and behavior, significance of the results (*p*-value), and the number of components used to generate the specified output. The data was analyzed using PLS regression in *scikit-learn* and cross-validated using LOOCV (Pedregosa et al., 2011). Significant correlations were those with a *p*-value below 0.05. The Benjamini Hochberg adjusted *p*-value adjusts for false discovery rate.

**FIGURE 3:**
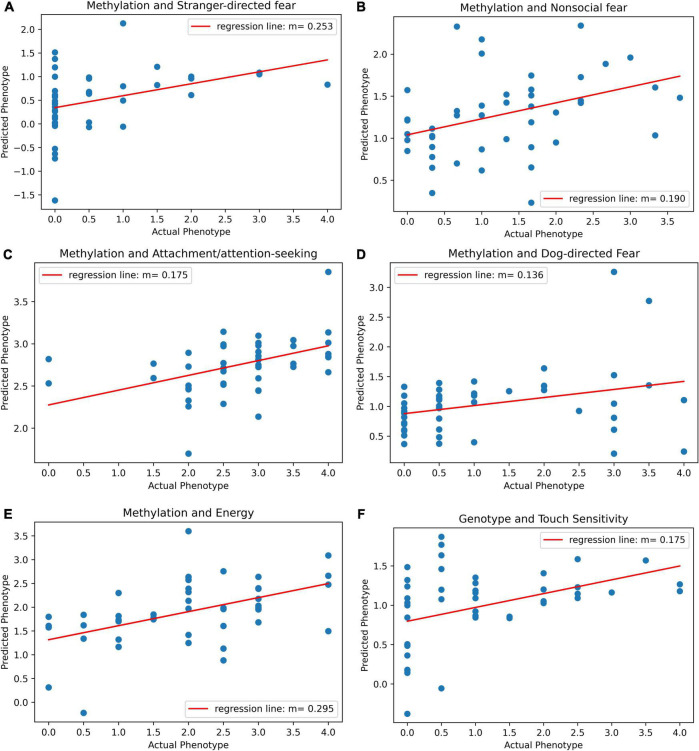
**(A)** Scatter plot of predicted vs. actual phenotype for stranger-directed fear. Actual stranger-directed fear values were recorded by the C-BARQ survey. Predicted phenotype scores were generated by PLS regression on methylation values. The plot specifies the slope of the best-fit line through the plotted points. **(B)** Scatter plot of predicted vs. actual phenotype for non-social fear. Actual non-social fear values were recorded by the C-BARQ survey. Predicted phenotype scores were generated by PLS regression on methylation values. The plot specifies the slope of the best-fit line through the plotted points. **(C)** Scatter plot of predicted vs. actual phenotype for attachment/attention-seeking. Actual attachment/attention-seeking values were recorded by the C-BARQ survey. Predicted phenotype scores were generated by PLS regression on methylation values. The plot specifies the slope of the best-fit line through the plotted points. **(D)** Scatter plot of predicted vs. actual phenotype for dog-directed fear. Actual dog-directed fear values were recorded by the C-BARQ survey. Predicted phenotype scores were generated by PLS regression on methylation values. The plot specifies the slope of the best-fit line through the plotted points. **(E)** Scatter plot of predicted vs. actual phenotype for energy. Actual energy values were recorded by the C-BARQ survey. Predicted phenotype scores were generated by PLS regression on methylation values. The plot specifies the slope of the best-fit line through the plotted points. **(F)** Scatter plot of predicted vs. actual phenotype for touch sensitivity. Actual touch sensitivity values were recorded by the C-BARQ survey. Predicted phenotype scores were generated by PLS regression on genotype values. The plot specifies the slope of the best-fit line through the plotted points.

Before adjusting for false discovery, five behavioral traits could be significantly predicted with epigenetic data. The most significant model was for energy (*r* = 0.49, *p* = 0.00053, *p*-adj = 0.00748). The PLS Regression model for energy was based on five components. The model can be visualized and its results are displayed as a scatterplot of predicted vs. actual energy phenotype value (Figure 3E). Models for other traits also produced significant results. The other traits that were significantly predicted were attachment/attention-seeking (*r* = 0.44, *p* = 0.002, *p*-adj = 0.0154), non-social fear (*r* = 0.38, *p* = 0.0099, *p*-adj = 0.0462), stranger-directed fear (*r* = 0.37, *p* = 0.011, *p*-adj = 0.0368), and dog-directed fear (*r* = 0.32, *p* = 0.033, *p*-adj = 0.0924) (Table 1). After adjusting for false discovery using The Benjamini Hochberg Procedure, energy, attachment/attention-seeking, non-social fear, and stranger-directed fear remained significant (*p*-adj < 0.05). Figures 3A–E displays a plot of predicted vs. actual phenotypes for each of the significantly correlated traits.

Touch sensitivity was the only trait that could be significantly predicted with genotype data (*r* = 0.41, *p* = 0.0047) (Table 1). However, after adjusting for false discovery touch sensitivity was no longer significant (*p*-adj = 0.0657) (Table 1). Figure 3F depicts the linear relationship between actual and predicted touch sensitivity behavioral values. No other phenotype was significantly predictable using genetic variants.

We used a biplot to inspect the loadings of the first two components in our PLS methylation analysis of behaviors (Supplementary Figure 1). Biplots show how each dependent variable contributes to the components of a regression model. In our biplots, we inspected each trait’s weighted contribution to the first two components of the PLS model. Traits are represented as points in a two-dimensional space: their x-coordinate indicating contribution to component 1 and y-coordinate indicating contribution to component 2. Supplementary Figure 1 showed that stranger-directed fear, non-social fear, attachment/attention-seeking, dog-directed fear, stranger-directed aggression, separation-related problems, chasing, and owner-directed aggression have greater weights in component 1 than component 2. Training difficulty, touch sensitivity, excitability, dog-directed aggression, energy, and familiar dog aggression have greater weights in component 2 than component 1. This analysis closely aligns with the trait-trait clustering revealed in Figure 2. All the traits with greater weights in component 1 except for owner-directed aggression were in Cluster 1 (Figure 2), implying a strong relationship between these traits.

### Age dependencies

Age was a possible confound of the association between methylation and behavioral phenotype. Therefore, for each of the four behaviors significantly associated with methylation we calculated age-adjusted methylation associations. We used the Pearson Correlational Coefficient to calculate associations between age and behavioral phenotypes (Harris et al., 2020). All four traits significantly correlated with age: energy (*r* = −0.443, *p*-value = 0.00205), attachment/attention-seeking (*r* = −0.369, *p*-value = 0.0116), non-social fear (*r* = −0.494, *p*-value = 0.000485), and stranger-directed fear (*r* = −0.348, *p*-value = 0.0181) (Supplementary Table 2). To differentiate the predictive power of age and methylation, we compared each model’s r-squared values for the significant traits. Of the four significant traits predicted using methylation data, only non-social fear was more significant in the age model (Supplementary Table 2). The methylation *R*_*M*_^2^ were higher in energy (*R*_*M*_^2^ = 0.241, *R*_*A*_^2^ = 0.197), attachment/attention-seeking (*R*_*M*_^2^ = 0.142, *R*_*A*_^2^ = 0.136), and stranger-directed fear (*R*_*M*_^2^ = 0.140, *R*_*A*_^2^ = 0.121) (Table 1 and Supplementary Table 2), suggesting that in most of the tested traits, methylation may contribute more significantly than age.

We also addressed age dependencies in the association of methylation and behavioral phenotypes by regressing age out of the traits. We used PLS regression to assess if methylation values were predictive of behavior trait residual values. Energy (*r* = 0.293, *p*-value = 0.0489) and stranger-directed fear (*r* = 0.363, *p*-value 0.0132) remained significantly associated with methylation in these models (Supplementary Table 3). The results of the age models indicate that age does contribute to the association between methylation and dog behavioral traits. However, there remain traits such as energy and stranger-directed fear that can be partially predicted by methylation alone. The persistence of these significant associations after adjusting for age indicates that methylation is a contributing factor to behavioral phenotypes.

### Association studies of individual loci

**FIGURE 4:**
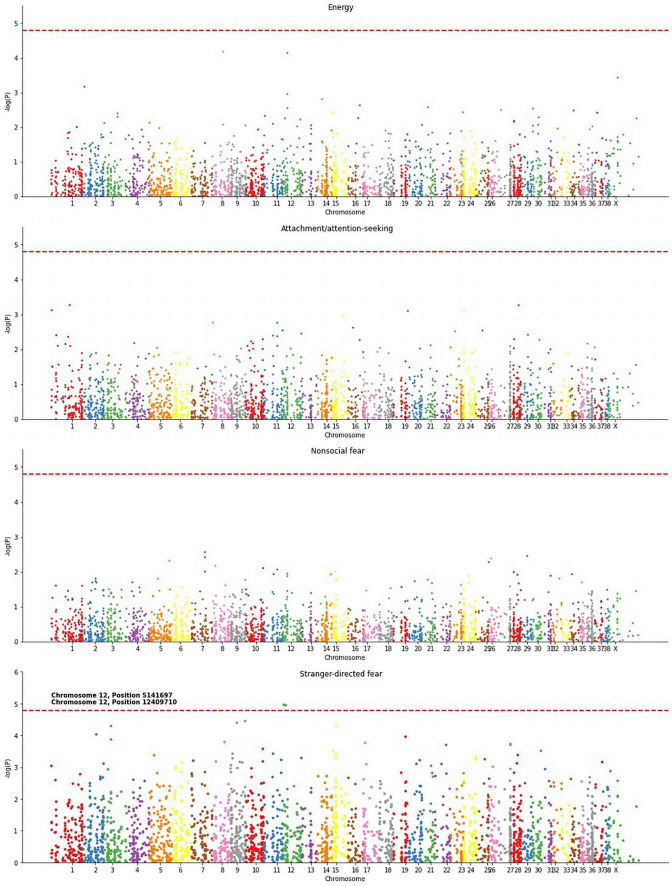
Manhattan plots for energy, attachment/attention-seeking, non-social fear, and stranger-directed fear. *P*-values, associated with an individual loci’s methylation and statistical significance in predicting behavior, were calculated using OLS Regression from *StatsModel* (Seabold and Perktold, 2010). Significant loci were determined using a Bonferroni threshold, enlarged, and labeled.

The significant correlations found between methylation and energy, attachment/attention-seeking, non-social fear, and stranger-directed fear suggest an association between DNA methylation and canine behaviors. We created Manhattan plots to identify specific loci whose age-adjusted methylation was associated with each of these four traits (Figure 4). The Manhattan plots in Figure 4 and Supplementary Figure 2 depict the association, measured as a -log10(*p*) value, of individual chromosome locations to a phenotype. Stranger-directed fear was the only trait out of the four analyzed that had individual loci significantly associated with its phenotype. Figure 4 shows two loci to be significantly associated with stranger-directed fear: chromosome 12 position 5141697 and chromosome 12 position 12409719 (Figure 4). A summary of the specific loci identified for each trait as well as genes at or near these loci is detailed in Table 2. We report loci with -log(*p*) values larger than the calculated Bonferroni Threshold contributing to each behavioral phenotype. The most significant locus [-log(p) = 4.96] is located within an exon of serine/arginine-rich splicing factor (*SRPK1*) (Kent et al., 2002). The second significant loci [-log(p) = 4.94] is within an exon on vascular endothelial growth factor A (*VEGFA*) (Kent et al., 2002).

**TABLE 2:** Association studies of individual Loci (Manhattan plots).

Trait	Chr	Position	Gene	-log(*p*)	Expression
Stranger-directed fear	12	5141697	*Serine/arginine-Rich Splicing Factor (SRPK1) (Non-dog RefSeq Gene)*	4.96	Broadly; majority in Whole Blood (GTEx Consortium, 2013)
Stranger-directed fear	12	12409710	*Vascular Endothelial Growth Factor A (VEGFA)*	4.94	Adipose (Subcutaneous), Adipose (Visceral), Artery (Aorta), Heart (Atrial Appendage), Prostate, Thyroid (GTEx Consortium, 2013)

Characterization of individual loci significant in phenotype analysis. Significant loci were generated through Manhattan plot analysis. Each loci is characterized by the trait it is significantly associated with, its closest gene, the -log(p) value of its association, and regions where it is most expressed. These genes at these loci were identified using the CanFam4 genome from the UCSC genome browser (Kent et al., 2002).

## Discussion

Behavioral variation is attributed to both genetic inheritance and environment; however, the respective influence of each of these factors on individual behaviors is a question that still requires clarification. Individual genes and genetic variants have been shown to contribute to behavioral phenotypes and behavioral disorders. Additionally, the regulation of genes by DNA methylation has also been associated with individual behaviors. In our analysis, we analyzed epigenetic and genetic influences on behavior. We hypothesized that our broad analysis of chromosome locations would reveal new associations between DNA methylation, genotype, and dog behaviors. Additionally, we predicted that comparing DNA methylation and genotype associations would reveal insights to their relative influence on shaping behavior development in dogs. After adjusting for false discovery rate and age bias, we found energy and stranger directed fear were significantly associated with DNA methylation. We also found that the limited panel of SNPs used in our analysis was not associated with behavioral traits. While these findings are preliminary, they support our hypothesis by demonstrating a link between DNA methylation and behavior.

Our analysis of the behavioral trait dataset revealed trait-breed and trait-trait correlations. In trait-breed analysis we found that dog breed did not have a strong influence on behavioral phenotypes. We also measured correlations between traits and found that the two most correlated traits were non-social fear and dog-directed fear. The analysis of behavioral phenotypes in Figure 2 revealed that they formed two clusters. Cluster 1 was composed of chasing, energy, separation-related problems, attachment/attention-seeking, stranger-directed aggression, stranger-directed fear, excitability, non-social fear, and dog-directed fear. Cluster 2 consisted of training difficulty, owner directed-aggression, touch sensitivity, dog-directed aggression, and familiar dog aggression. We expect energy related traits such as energy, chasing, and excitability to be more related than hostility traits such as owner-directed aggression, touch sensitivity, or dog-directed aggression. Previous research has suggested that traits such as fear and aggression in dogs are correlated with one another (Zapata et al., 2016), but our cluster analysis reveals low correlation between dog-directed aggression and dog-directed fear. This is likely due to the low sample size and unusual breed composition in our study.

Additionally, we did not find that dogs of the same breed cluster together based on their behaviors (Figure 1). The behavioral variance of Australian Shepherds, the most abundant breed, was greater than the behavioral variance of our entire sample. The high variance between breed and phenotype suggests that there may be a broader range of behaviors within each breed than initially expected. It is important to note that this analysis was completed with a small sample size and should be confirmed with a larger sample size.

To analyze epigenetic and genetic components of behavioral traits, we used PLS regression models. PLS accounts for covariance between independent and dependent variables and allows high dimensional data to be embedded into lower dimensional components. PLS is particularly useful in scenarios where there exists many dependent variables and many correlated independent variables. We also pursued PLS regression because it is well established within epigenetics research. For example, a recent study which analyzed 1,982 probes trained a PLS model on a mix of schizophrenic and non-psychiatric patients and determined that symptoms of schizophrenia are influenced by unique methylation at correlated regions of systemic interindividual variation (Gunasekara et al., 2021).

Examining DNA methylation across the 46 dogs in our sample revealed new information on the epigenetic basis of dog behaviors. PLS regression analysis revealed energy, attachment/attention-seeking, non-social fear, stranger-directed fear, and dog-directed fear to be significantly predictable using methylation data. Of these traits energy, attachment/attention-seeking, non-social fear, and stranger-directed fear had significant adjusted *p*-values. Our findings of significant methylation-behavior associations in energy, attachment/attention-seeking, non-social fear, and stranger-directed fear support previous work that found dog social behavior to be significantly associated with promoter methylation (Cimarelli et al., 2017). These findings support our hypothesis that analyzing a broad sample of chromosome locations reveals new information about the genetic basis of behavioral development. Earlier studies in worker bees found preliminary results that social experiences and behaviors impact the epigenome, concluding that further work needs to be done to analyze epigenetic regulation of behavioral development (Rehan et al., 2016). The present study can serve as a proof of concept for using bisulfite sequencing to study the epigenetic basis of such behaviors across species. However, the age bias and low power of our study should be noted. Our findings therefore require further testing to validate our results.

Our analysis of the genotype dataset did not reveal any significant associations between genotype and behavioral traits. Touch sensitivity was shown to be significantly predictable using SNPs. However, no trait had significant adjusted *p*-values after correcting for multiple testing using the Benjamini Hochberg Procedure. The lack of genotype associations compared to DNA methylation associations could indicate that methylation plays a larger role in influencing the dog behaviors examined in this study. However, the small selection of SNPs included in genotype analysis precludes this conclusion. The lack of significant associations between behaviors and genotypes may be due to the fact that we only measured a small fraction of SNPs using our targeted bisulfite assay. There were only 930 specific SNPs used in analysis and these sites were contained within the regions that we captured using our probe pool. In previous genome wide association studies (GWAS), researchers have found SNPs to be associated with canine behaviors. A previous GWAS study in beagles found five candidate genes to be associated with human-directed social behaviors in dogs (Persson et al., 2016). Across species, GWAS in honeybees have found four candidate genes to be associated with defense behaviors (Spötter et al., 2016). Therefore, it is certain that other SNPs within the dog genome could contribute more significantly to behavior. Future studies should incorporate a greater selection of SNPs to better capture genotype influence in behavior analysis.

The associations between DNA methylation and behavioral traits suggested that differences in gene expression could contribute to behavioral differences between dogs. Therefore, we sought to examine specific loci whose methylation was significantly associated with behavioral traits. We created Manhattan Plots for each of the four traits that were significantly associated with DNA methylation in our initial analysis. These Manhattan plots displayed the significance of individual associations between one behavioral trait and each chromosome position included in our methylation matrix (Figure 4 and Supplementary Figure 2).

The study of associations between single loci and traits revealed sites that were significantly associated with each of the four phenotypes we analyzed (Figure 4). The genes adjacent to these significant sites provided interesting hypotheses about possible mechanisms involved in behavioral regulation by gene expression (Table 2). The loci significantly associated with stranger-directed fear span internal exons of *SRPK1* and *VEGFA* which have a CpG island at its 5′ end. These genes are highly conserved within many mammals, suggesting that further research should be conducted. The former, *SRPK1*, has been found to have high expression in neurons in the brain (Mytilinaios et al., 2012). It is plausible that in the brain *SRPK1* regulates splicing of neurons and neuronal differentiation (Mytilinaios et al., 2012). Perhaps its associations with neuron development impact behavioral development. The latter, *VEGFA*, has been found to have neuroprotective effects in the central nervous system through protecting neurons from degradation and cell death (Axelsen and Woldbye, 2018). *VEGFA* has been proposed as a plausible gene therapy for Parkinson’s disease patients because of its role in the nervous system (Axelsen and Woldbye, 2018). Since *VEGFA* helps guard against neuron dysfunction, perhaps regulation of this gene alters dog behaviors.

Though they did not meet the Bonferroni threshold, it is worth noting that several loci in chromosome 2 were suggestive of methylation association with touch sensitivity (Supplementary Figure 2). Chromosome 2 positions 51331684, 52739194, 52739210 are located downstream of MAST4 and within an intron of PIK3R1. Further studies should explore these genes and loci to better understand methylation and its association with touch sensitivity.

Thus, we were able to identify loci whose methylation is significantly correlated with behavioral phenotypes and are located near genes that impact the nervous system or behavior. It is likely that significantly associated loci are located at the promoter, enhancer, or regulatory element of their adjacent genes, although these regions are not yet well described in the dog genome. We therefore hypothesize that differential methylation of these loci could impact gene expression and dog behavior.

There were several limitations in our study. The low sample size of 46 dogs reduces the power of this study. Future work should be done with a larger sample size to validate our findings. Additionally, the owner’s bias in reporting has an effect on the dogs’ behavior data. If the owner has known the dog for longer, it is likely they have a better understanding of their dog’s behavior. As we have shown, a dog’s age can also influence the behavior. This effect is likely amplified by our study design. The limited number of probes in this study were selected by Rubbi et al. (2022) and included loci whose methylation was associated with age. The presence of such ascertainment bias required correction for the age association within all of our analysis. In addition, the strong linkage disequilibrium in dogs paired with the low number of SNPs in our study make it possible that mapped signals are detected because they are in LD with the true functional variant. Again, future studies with a broader SNP panel can further explore this issue.

Our initial analysis found evidence that behavioral phenotypes can be predicted from methylation data. However, the influence of age may preclude our ability to confidently conclude that energy, attachment/attention-seeking, non-social fear, and stranger-directed fear are significantly predictable by DNA methylation profiles. Across species, aging is known to impact animal behaviors. A study in mice found significant differences in behavior between age groups with older mice exhibiting decreased movement and social behaviors, and increased anxiety (Shoji et al., 2016). We used two different methods to determine the effect of age on behavior. In both methods energy and stranger-directed fear remained significantly associated with DNA methylation after adjusting for age bias (Supplementary Tables 2, 3). This suggests that the associations between DNA methylation and behaviors are not solely due to age associated changes in DNA methylation. The persistence of energy and stranger-directed fear associations adds confidence to our analysis. This finding aligns with previous research that links epigenetic mechanisms regulating translational access to genes and fear memory formation (such as in post-traumatic stress disorder or PTSD). Previous studies have shown that the inhibition of DNA methyltransferase (DNMT) activity impedes hippocampal-dependent memory formation processes, such as contextual fear memory creation (Miller and Sweatt, 2007; Lubin et al., 2008; Feng et al., 2010; Han et al., 2010). Similarly, other research has found that auditory fear conditioning is associated with an increase in DNMT3a protein within the lateral amygdala suggesting that DNMT activity is necessary for fear memory consolidation (Monsey et al., 2011). These findings implicate DNMT activity as a crucial part of synaptic plasticity and fear memory synthesis. Thus, it is not surprising that our study found energy and stranger-directed fear to remain significant after correcting for age bias. We can propose that a few traits are predictable by DNA methylation and that the associations between DNA methylation and behaviors are not solely due to DNA methylation age associations. Future research projects should control for age in subject recruitment to strengthen the genetic association of behavior.

In conclusion, we propose that energy and stranger-directed fear are partially predictable by DNA methylation in dogs. These behavioral associations are of interest because they provide evidence that changes in methylation can impact personalities, energy levels, and other aspects of behavior. Since methylation is dynamic and can be influenced by environmental factors, in the future we might be able to modify canine behavior by modulating these factors. Our findings should motivate additional studies to identify the epigenetic basis of behaviors across species. Future studies could utilize probe panels that capture more loci to better study the impact of methylation on behavior. Additionally, more behavioral traits such as dog herding and ball retrieving could be analyzed as these behaviors are unique to dogs. Since dogs are a valuable model organism for humans, it is plausible that similar associations may exist between epigenetics and human behavior.

## Data availability statement

The datasets presented in this study can be found in online repositories. The names of the repository/repositories and accession number(s) can be found in the article/Supplementary material.

## Ethics statement

Ethical review and approval was not required for the animal study because samples from canines were collected with ethical review in a previous study cited within the manuscript. Our study collected behavior surveys from dog owners. Animal ethics committee approval was not required. Written informed consent was obtained from the owners for the participation of their animals in this study.

## Author contributions

AS wrote the Abstract, Introduction, Methods, Results, Discussion, compiled References, and ran regression models. NB wrote the Introduction, Methods, Results, and created summary figures. MP led at the research time during the manuscript’s development and provided guidance to the first authors. BV reviewed the manuscript, provided feedback, and proposed future directions. All authors contributed to the article and approved the submitted version.

## Supplementary material

Supplementary Data Sheet 1 Supplements.

Supplementary Data Sheet 2 Supplementary file 1.

Supplementary Data Sheet 3 Supplementary file 2.

Supplementary Data Sheet 4 Supplementary file 3.
