# Supplementary material for: Association of DNA methylation with energy and fear-related behaviors in canines
Source: Front Psychol. 2022 Dec 14;13:1025494. doi: 10.3389/fpsyg.2022.1025494 (PMC9794564; doi:10.3389/fpsyg.2022.1025494)
Supplement: Supplementary Data Sheet 1 — Supplements. [file Data_Sheet_1.docx]

Supplementary Material

# Supplementary Figure 1

Biplot of the loadings of the top 2 PLS components from PLS behavioral analysis. Each behavior is a point in 2-dimensional space where its coordinates represent the amount of weight that the trait has on the component. The x-axis represents influence on component 1 while the y-axis represents influence on component 2.

**
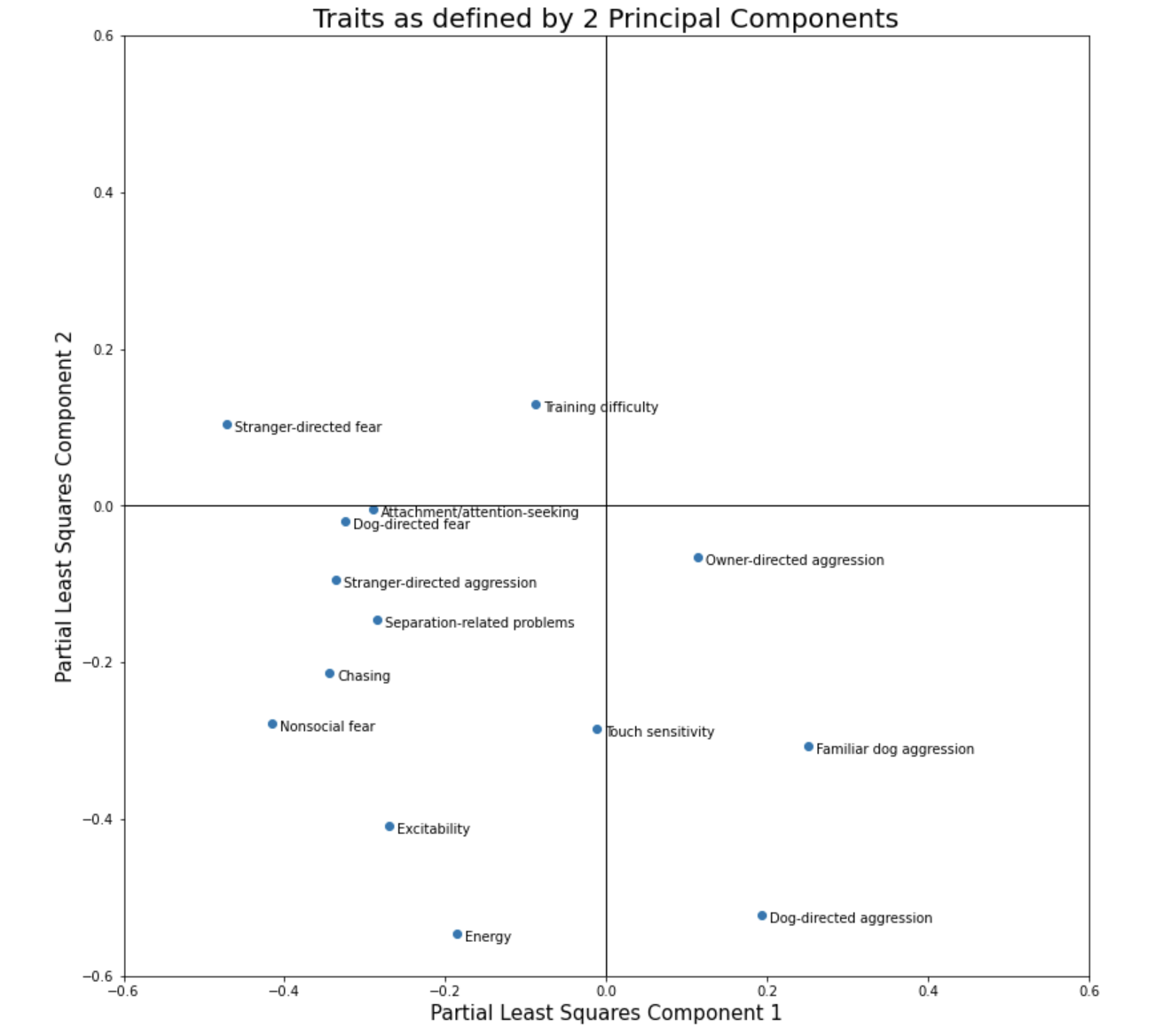
**

# Supplementary Figure 2

 Manhattan plots for excitability, stranger-directed aggression, owner-directed aggression, dog-directed aggression, familiar dog aggression, dog-directed fear, touch sensitivity, separation-related problems, training difficulty, and chasing. P-values, associated with an individual loci’s methylation and statistical significance in predicting behavior, were calculated using OLS Regression from *StatsModel* (Seabold and Perktold, 2010). Significant loci were determined using a Bonferroni threshold.

**
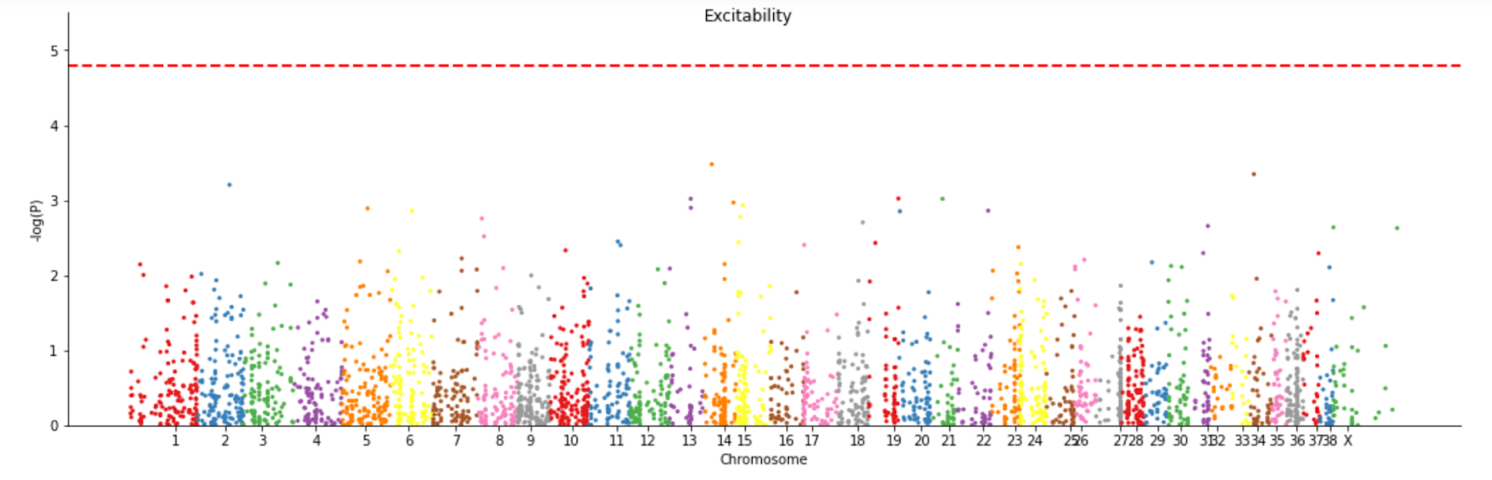
**

**
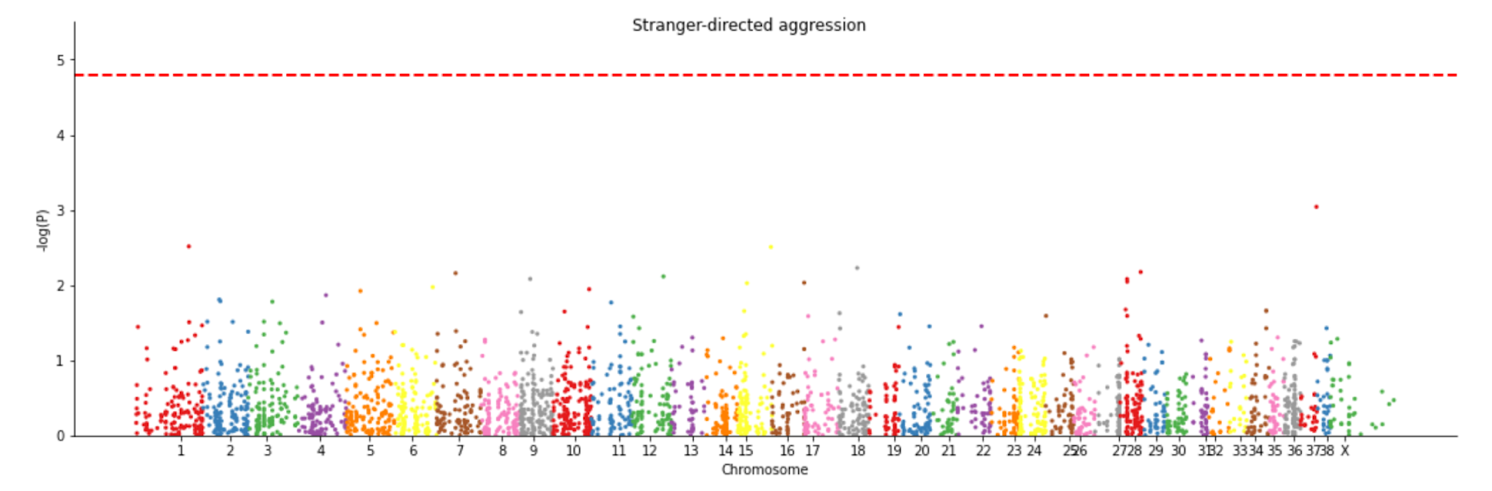
**

**
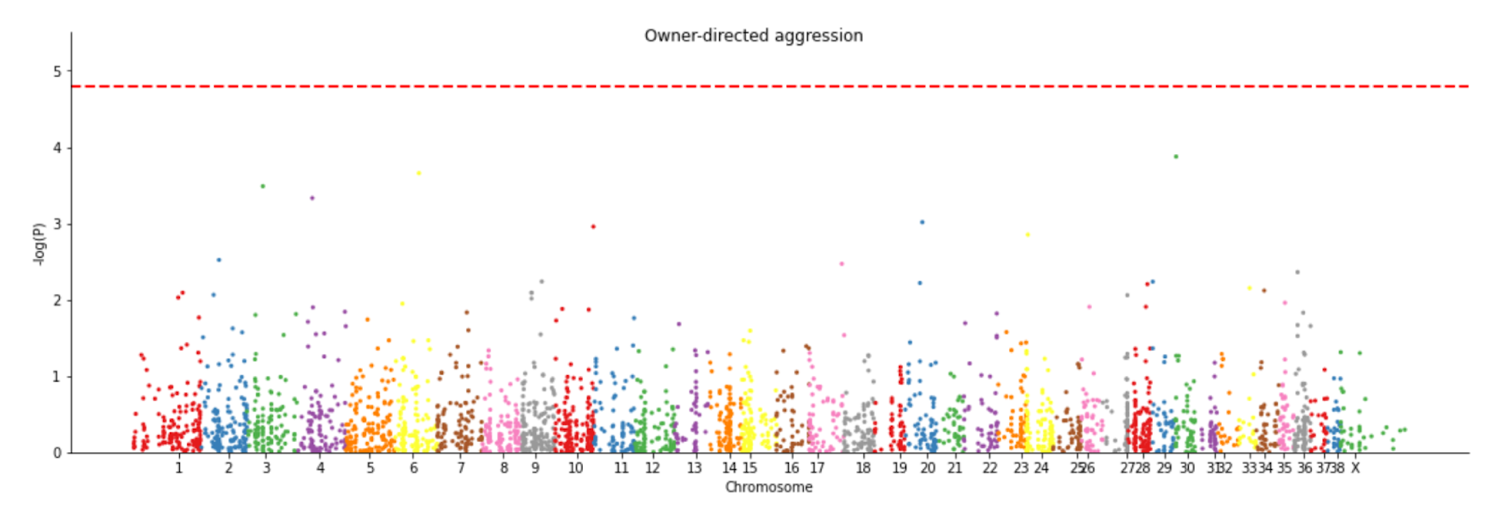
**

**
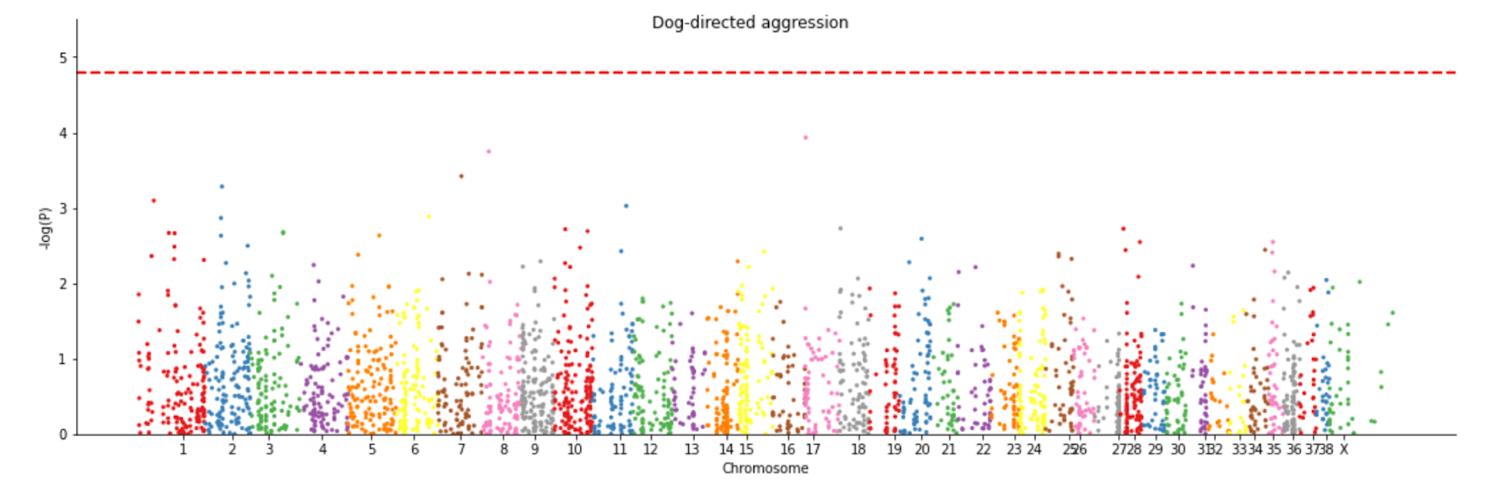
**

**
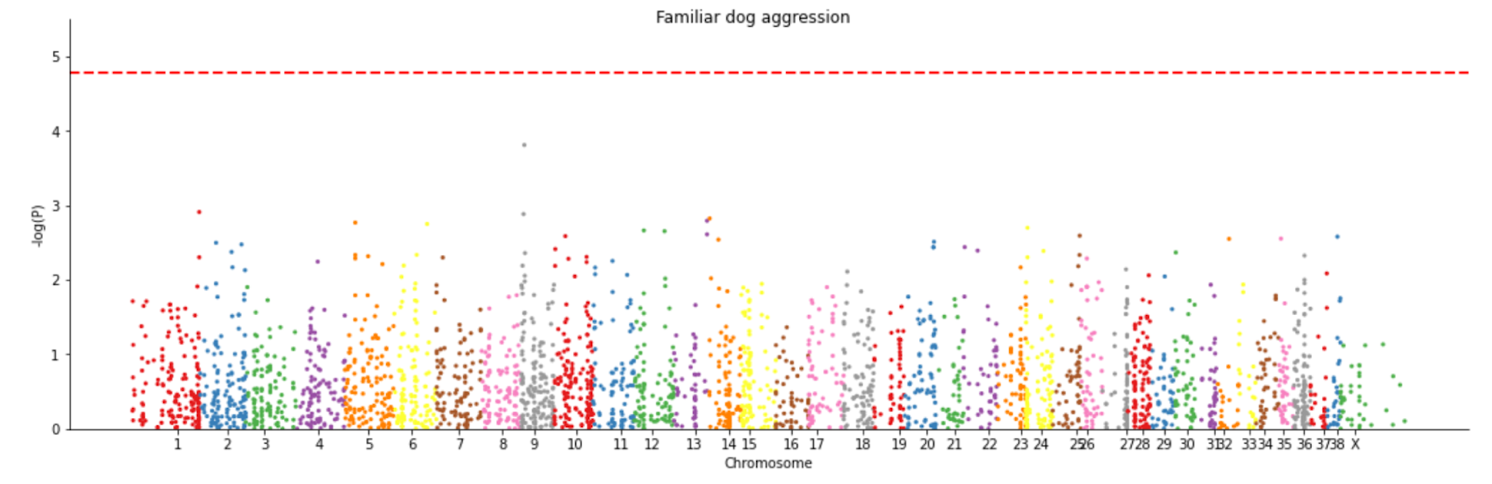
**

**
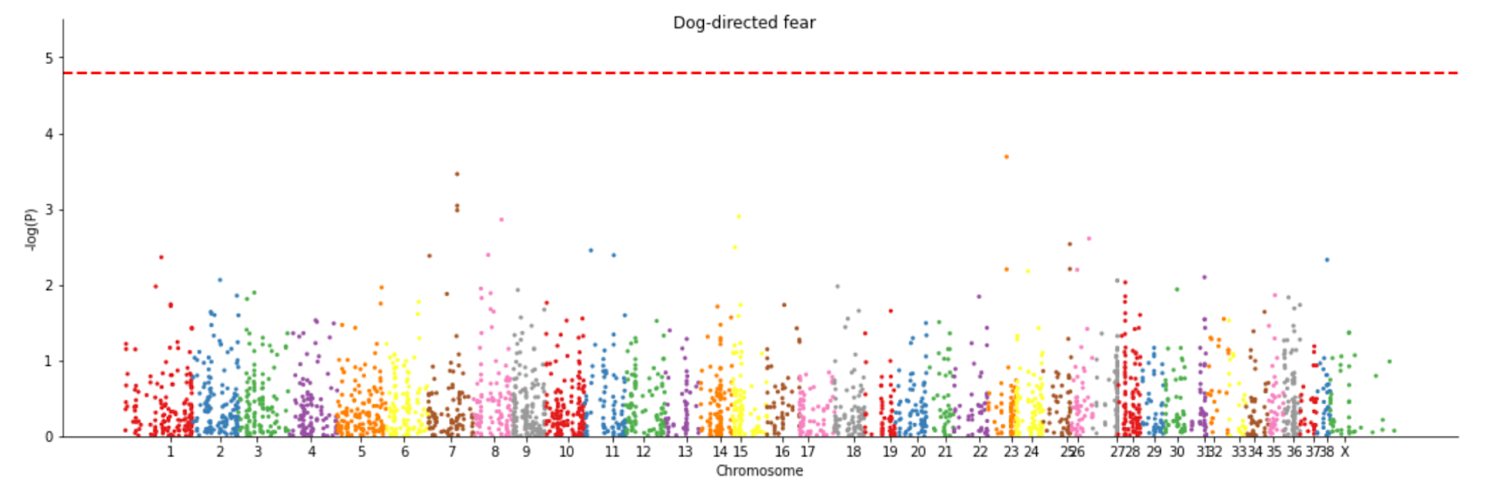
**

**
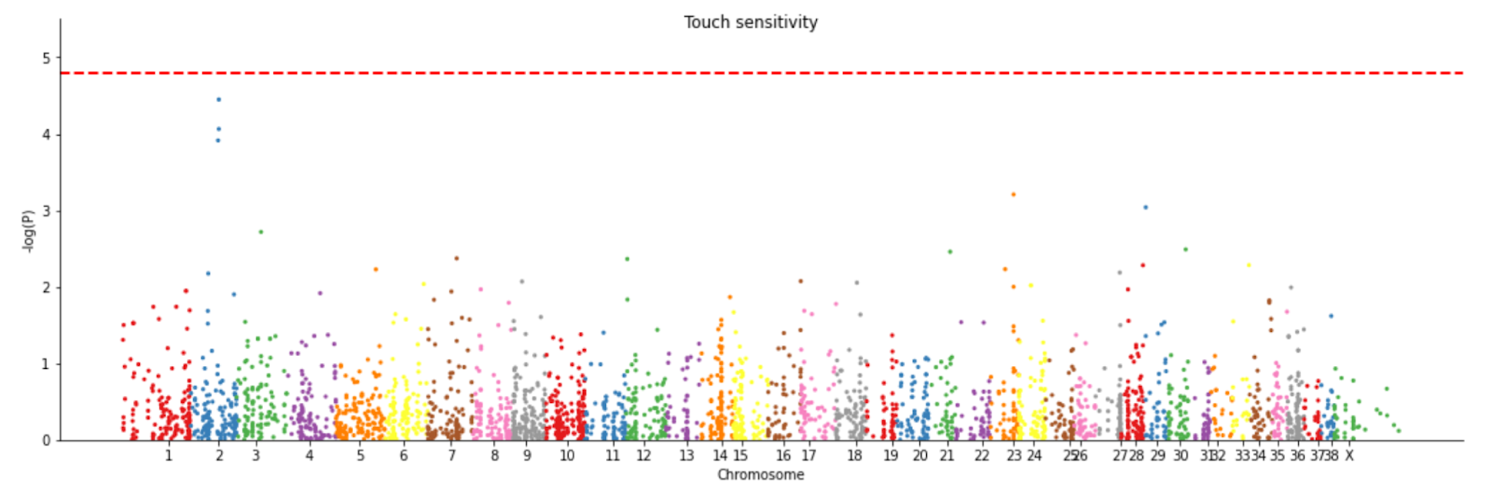
**

**
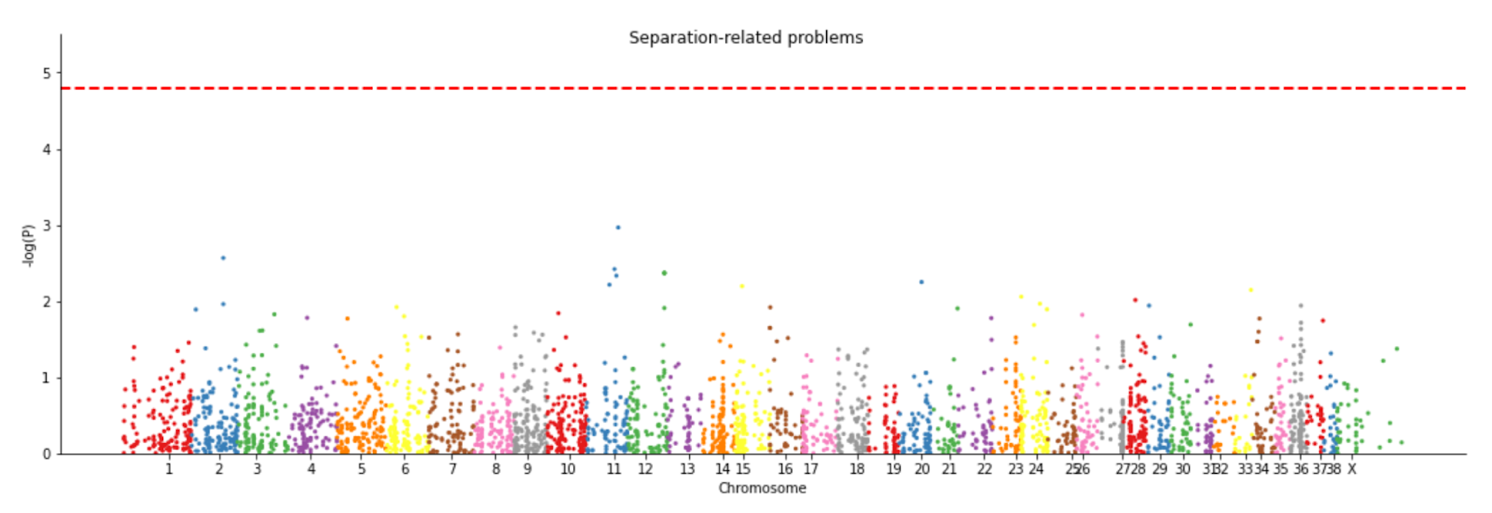
**

**
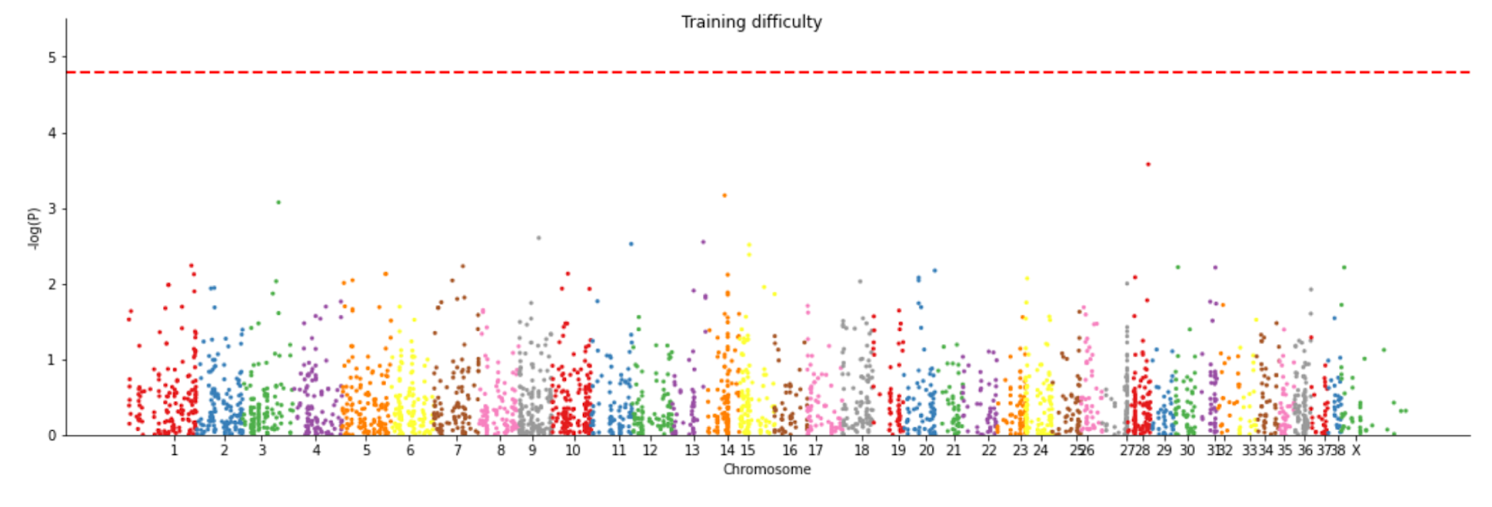
**

**
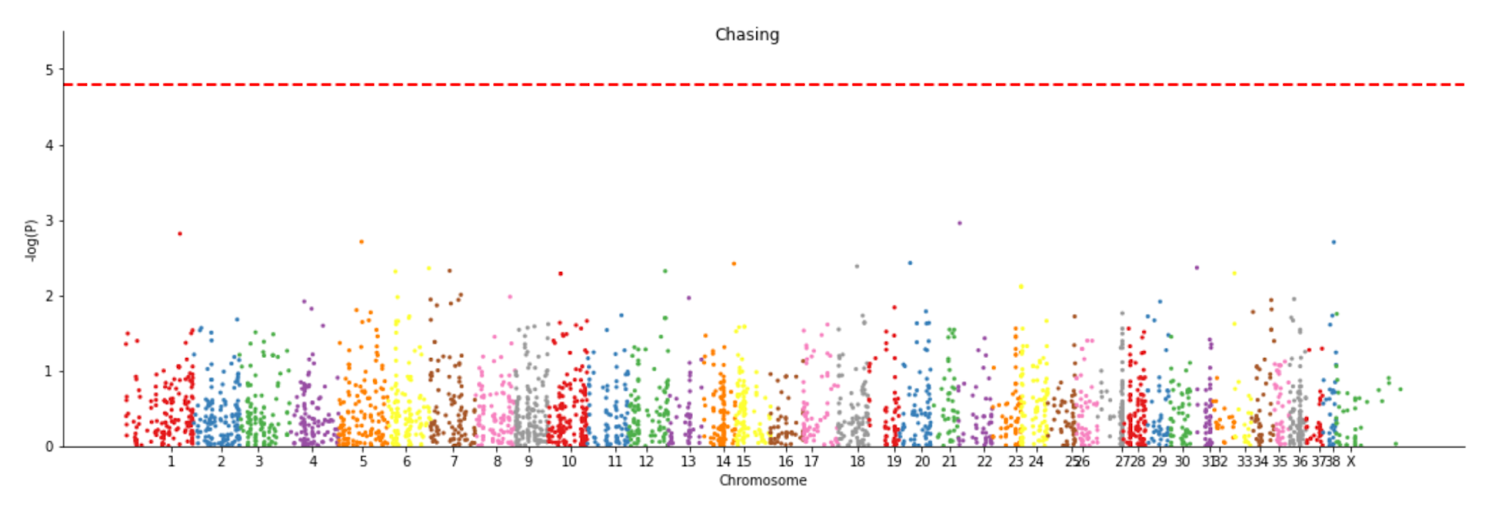
**

# Supplementary Table 1

Description of each phenotypic trait used in the dog behavioral analysis. The descriptions were provided by the C-BARQ website. The definitions are each trait are used to frame questions for owners to classify their dogs’ behaviors.

| Trait | CBAR-Q Description |
| --- | --- |
| Stranger-directed aggression | Threatening or hostile responses to strangers approaching or invading the dog's or owner's personal space, territory, or home range. |
| Owner-directed aggression | Threatening or hostile responses to the owner or other members of the household when challenged, manhandled, stared at, stepped over, or when approached while in possession of food or objects. |
| Dog-directed aggression | Threatening or hostile responses when approached by unfamiliar dogs |
| Familiar dog aggression | Threatening or hostile responses to other familiar dogs in the same household. |
| Stranger-directed fear | Fearful or wary responses when approached by strangers. |
| Nonsocial fear | Fearful or wary responses to sudden or loud noises, traffic, and unfamiliar objects and situations. |
| Dog-directed fear | Fearful or wary responses when approached by unfamiliar dogs. |
| Separation-related problems | Vocalizing and/or destructiveness when separated from the owner, often accompanied or preceded by behavioral and autonomic signs of anxiety including restlessness, loss of appetite, trembling, and excessive salivation. |
| Attachment and attention-seeking | Maintaining close proximity to the owner or other members of the household, soliciting affection or attention, and displaying agitation when the owner gives attention to third parties. |
| Training difficulty | Willingness to attend to the owner, obey simple commands, learn quickly, fetch objects, respond positively to correction, and ignore distracting stimuli. |
| Chasing | Chasing cats, birds, and/or other small animals, given the opportunity. |
| Excitability | Displaying strong reactions to potentially exciting or arousing events, such as going for walks or car trips, doorbells, arrival of visitors, and the owner arriving home; has difficulty settling down after such events. |
| Touch Sensitivity | Fearful or wary responses to potentially painful procedures, including bathing, grooming, nail-clipping, and veterinary examinations. |
| Energy | Energetic, “always on the go”, and/or playful. |

# Supplementary Table 2

Summary of the correlations between age and the 4 behavioral traits. Each correlation is calculated using correlation coefficients between reported age and trait values.

| Trait | Correlation | R^2^ | P-value |
| --- | --- | --- | --- |
| Stranger-directed fear | -0.348 | 0.121 | 0.0181 |
| Nonsocial fear | -0.494 | 0.244 | 0.000485 |
| Attachment/attention-seeking | -0.369 | 0.136 | 0.0116 |
| Energy | -0.443 | 0.197 | 0.00205 |

# Supplementary Table 3

Summary of the statistics of the PLS regression results between methylation and age-adjusted behaviors. Age-adjusted behaviors were determined by the difference between behavior values predicted by age and actual behaviors. P-value, correlation, and number of components are specified for each trait.

| **Traits** | **Age-adjusted Correlation Coefficient**  **(R-value)** | **P-value** | **Number of Components in Regression Model** |
| --- | --- | --- | --- |
| Stranger-directed fear | 0.363 | 0.0132 | 1 |
| Energy | 0.293 | 0.0485 | 5 |
| Attachment/ attention-seeking | 0.290 | 0.0504 | 6 |
| Nonsocial fear | 0.121 | 0.424 | 5 |
