# Supplementary material for: Association of DNA methylation with energy and fear-related behaviors in canines
Source: Front Psychol. 2022 Dec 14;13:1025494. doi: 10.3389/fpsyg.2022.1025494 (PMC9794564; doi:10.3389/fpsyg.2022.1025494)
Supplement: Supplementary Data Sheet 2 — Supplementary file 1. [file Data_Sheet_2.pdf]

Dog's name: ______________________________

Canine Behavioral Assessment & Research Questionnaire (short version)

SECTION 1: Excitability

INSTRUCTIONS: Some dogs show little reaction to exciting events, while others become highly excited at the slightest novelty. By circling a number on the following 5-point scales (0=Calm, 4=Extremely excitable), please indicate your own dog's recent tendency to become excitable in the following circumstances **(please circle only one number)**:

1. Just before being taken for a walk.

Mild—Moderate excitability

Calm: little or no special reaction 0..............1..............2..............3..............4 **Extremely excitable:** over-reacts, hard to calm down.

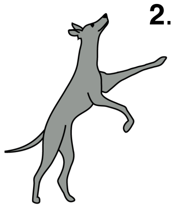

2. Just before being taken on a car trip.

Mild—Moderate excitability

Calm: little or no special reaction 0..............1..............2..............3..............4 **Extremely excitable:** over-reacts, hard to calm down.

SECTION 2: Aggression

INSTRUCTIONS: Most dogs display aggressive behavior from time to time—e.g. barking, growling, baring teeth, snapping, etc. By circling a number on the following 5-point scales (0= No aggression, 4= Serious aggression), please indicate your own dog's recent tendency to display aggressive behavior in each of the following circumstances **(please circle only one number)**:

3. When approached directly by an unfamiliar **person** while being walked/exercised on a leash.

Moderate aggression: growling/barking—baring teeth

No aggression: No visible signs of aggression 0..............1..............2..............3.............4 **Serious aggression:** Snaps, bites or attempts to bite.

4. When toys, bones or other objects are taken away by a household member.

Moderate aggression: growling/barking—baring teeth

No aggression: No visible signs of aggression 0..............1..............2..............3.............4 **Serious aggression:** Snaps, bites or attempts to bite.

5. When approached directly by a household member while s/he (the dog) is eating.

Moderate aggression: growling/barking—baring teeth

No aggression: No visible signs of aggression 0..............1..............2..............3.............4 **Serious aggression:** Snaps, bites or attempts to bite.

© 2018 James A. Serpell, University of Pennsylvania

6. When mailmen or other delivery workers approach your home.

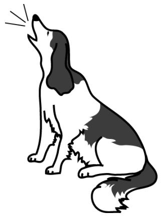

No aggression: No visible signs of aggression	Moderate aggression: growling/barking—baring teeth	Serious aggression: Snaps, bites or attempts to bite.
	0..............1..............2..............3.............4	

7. When his/her food is taken away by a household member.

No aggression: No visible signs of aggression	Moderate aggression: growling/barking—baring teeth	Serious aggression: Snaps, bites or attempts to bite.
	0..............1..............2..............3.............4	

8. When approached directly by an unfamiliar **dog** while being walked/exercised on a leash.

No aggression: No visible signs of aggression	Moderate aggression: growling/barking—baring teeth	Serious aggression: Snaps, bites or attempts to bite.
	0..............1..............2..............3.............4	

9. When strangers walk past your home when your dog is outside or in the yard.

No aggression: No visible signs of aggression	Moderate aggression: growling/barking—baring teeth	Serious aggression: Snaps, bites or attempts to bite.
	0..............1..............2..............3.............4	

10. When barked, growled, or lunged at by another (unfamiliar) dog.

No aggression: No visible signs of aggression	Moderate aggression: growling/barking—baring teeth	Serious aggression: Snaps, bites or attempts to bite.
	0..............1..............2..............3.............4	

11. When approached while eating by another (familiar) household **dog** (leave blank if no other dogs).

No aggression: No visible signs of aggression	Moderate aggression: growling/barking—baring teeth	Serious aggression: Snaps, bites or attempts to bite.
	0..............1..............2..............3.............4	

12. When approached while playing with/chewing a favorite toy, bone, object, etc., by another (familiar) household **dog** (leave blank if no other dogs).

No aggression: No visible signs of aggression	Moderate aggression: growling/barking—baring teeth	Serious aggression: Snaps, bites or attempts to bite.
	0..............1..............2..............3.............4	

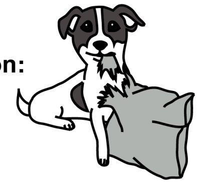

SECTION 3: Fear and Anxiety

INSTRUCTIONS: Dogs often show signs of anxiety or fear when exposed to particular sounds, objects, persons or situations—e.g. crouching or cringing with tail tucked between the legs; whimpering or whining, freezing, trembling, or attempting to escape or hide. Using the following 5-point scales (0=No fear, 4=Extreme fear), please indicate your own dog's recent tendency to display fearful behavior in the following circumstances **(please circle only one number)**:

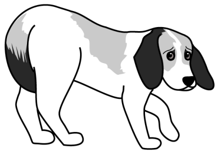

13. When approached directly by an unfamiliar person while away from your home.

No fear/anxiety: No visible signs of fear 0..............1..............2..............3..............4 **Extreme fear:** cowers; retreats or hides, etc.
Mild—Moderate fear/anxiety

14. In response to sudden or loud noises (e.g. thunder, vacuum cleaner, car backfire, road drills, objects being dropped, etc.).

No fear/anxiety: No visible signs of fear 0..............1..............2..............3..............4 **Extreme fear:** cowers; retreats or hides, etc.
Mild—Moderate fear/anxiety

15. When an unfamiliar person tries to touch or pet the dog.

No fear/anxiety: No visible signs of fear 0..............1..............2..............3..............4 **Extreme fear:** cowers; retreats or hides, etc.
Mild—Moderate fear/anxiety

16. In response to strange or unfamiliar objects on or near the sidewalk (e.g. plastic trash bags, leaves, litter, flags flapping, etc.

No fear/anxiety: No visible signs of fear 0..............1..............2..............3..............4 **Extreme fear:** cowers; retreats or hides, etc.
Mild—Moderate fear/anxiety

17. When approached directly by an unfamiliar dog.

No fear/anxiety: No visible signs of fear 0..............1..............2..............3..............4 **Extreme fear:** cowers; retreats or hides, etc.
Mild—Moderate fear/anxiety

18. When first exposed to unfamiliar situations (e.g. first car trip, first time in elevator, first visit to veterinarian, etc.).

No fear/anxiety: No visible signs of fear 0..............1..............2..............3..............4 **Extreme fear:** cowers; retreats or hides, etc.
Mild—Moderate fear/anxiety

19. When barked, growled, or lunged at by an unfamiliar dog.

No fear/anxiety: No visible signs of fear 0..............1..............2..............3..............4 **Extreme fear:** cowers; retreats or hides, etc.
Mild—Moderate fear/anxiety

© James A. Serpell, University of Pennsylvania

20. When having nails clipped by a household member.

No fear/anxiety: No visible signs of fear	Mild—Moderate fear/anxiety	Extreme fear: cowers; retreats or hides, etc.
	0..............1..............2..............3..............4	

21. When groomed or bathed by a household member.

No fear/anxiety: No visible signs of fear	Mild—Moderate fear/anxiety	Extreme fear: cowers; retreats or hides, etc.
	0..............1..............2..............3..............4	

SECTION 4: Separation-related behavior.

INSTRUCTIONS: Some dogs show signs of anxiety when left alone, even for short periods of time. Thinking back over the recent past, how often has your dog shown each of the following signs of anxiety when left, or about to be left, on its own **(please check only one box per question)**:

	Never	Seldom	Sometimes	Usually	Always
**22.** Restlessness/agitation/pacing.	☐	☐	☐	☐	☐
**23.** Barking or whining.	☐	☐	☐	☐	☐
**24.** Chewing/scratching at doors, floor, windows, curtains, etc.	☐	☐	☐	☐	☐

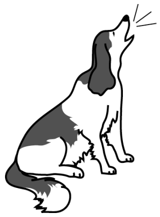

SECTION 5: Attachment and Attention-seeking.

INSTRUCTIONS: Most dogs are strongly attached to their people, and some demand a great deal of attention and affection from them. Thinking back over the recent past, how often has your dog shown each of the following signs of attachment or attention-seeking **(please check only one box per question)**:

	Never	Seldom	Sometimes	Usually	Always
**25.** Tends to follow you (or other members of the household) about the house, from room to room.	☐	☐	☐	☐	☐
**26.** Tends to sit close to, or in contact with, you (or others) when you are sitting down	☐	☐	☐	☐	☐

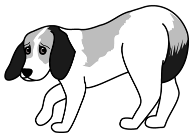

© James A. Serpell, University of Pennsylvania

SECTION 6: Training and obedience

INSTRUCTIONS: Some dogs are more obedient and trainable than others. By checking the appropriate boxes, please indicate how trainable or obedient your dog has been in each of the following situations in the recent past **(please check only one box per question)**:

	Never	Seldom	Sometimes	Usually	Always
**27.** Obeys a "sit" command immediately.	☐	☐	☐	☐	☐
**28.** Obeys a "stay" command immediately.	☐	☐	☐	☐	☐
**29.** Easily distracted by interesting sights, sounds or smells.	☐	☐	☐	☐	☐

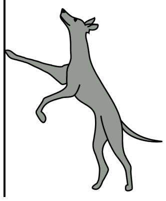

SECTION 7: Miscellaneous problems

INSTRUCTIONS: Dogs display a wide range of miscellaneous behavior problems in addition to those already covered by this questionnaire. Thinking back over the recent past, please indicate how often your dog has shown any of the following behaviors **(please check only one box per question)**:

	Never	Seldom	Sometimes	Usually	Always
**30.** Chases or would chase birds, given the chance.	☐	☐	☐	☐	☐
**31.** Chases or would chase squirrels, rabbits, etc., given the chance.	☐	☐	☐	☐	☐
**32.** Escapes or would escape from home or yard, given the chance.	☐	☐	☐	☐	☐
**33.** Chews inappropriate objects.	☐	☐	☐	☐	☐
**34.** Pulls excessively hard when on the leash.	☐	☐	☐	☐	☐
**35.** Urinates against objects/ furnishings in your home.	☐	☐	☐	☐	☐
**36**. Urinates when left alone at night, or during the daytime.	☐	☐	☐	☐	☐
**37**. Defecates when left alone at night, or during the daytime.	☐	☐	☐	☐	☐

© James A. Serpell, University of Pennsylvania

**38.** Hyperactive, restless, has trouble settling down.	☐	☐	☐	☐	☐
**39.** Playful, puppyish, boisterous.	☐	☐	☐	☐	☐
**40.** Active, energetic, always on the go.	☐	☐	☐	☐	☐
**41.** Chases own tail/hind end.	☐	☐	☐	☐	☐
**42**. Barks persistently when alarmed or excited.	☐	☐	☐	☐	☐

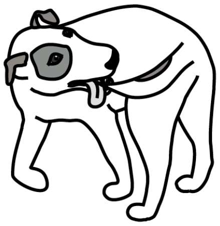

Thank you for providing this helpful information!

© James A. Serpell, University of Pennsylvania
