# Supplementary material for: Association of DNA methylation with energy and fear-related behaviors in canines
Source: Front Psychol. 2022 Dec 14;13:1025494. doi: 10.3389/fpsyg.2022.1025494 (PMC9794564; doi:10.3389/fpsyg.2022.1025494)
Supplement: Supplementary Data Sheet 3 — Supplementary file 2. [file Data_Sheet_3.pdf]

C-BARQ short version scoring method

The C-BARQ[42] provides a set of quantitative scores for the following fourteen different subscales or categories of behavior:

1. ***Excitability***
2. ***Stranger-directed aggression***
3. ***Owner-directed aggression***
4. ***Dog-directed aggression***
5. ***Familiar dog aggression***
6. ***Stranger-directed fear***
7. ***Nonsocial fear***
8. ***Dog-directed fear***
9. ***Touch sensitivity***
10. ***Separation-related behavior***
11. ***Attachment and attention-seeking***
12. ***Trainability***
13. ***Chasing***
14. ***Energy level***

The C-BARQ[42] also provides useful information on the occurrence of an additional 9 miscellaneous behavior problems ranging from escaping and roaming to persistent barking.

Each subscale is represented by a small number of 5-point scales (questions). Some are graduated scales that measure severity of particular behaviors (e.g. aggression) and are numbered from 0–4 in the questionnaire. The remainder are frequency scales which should be scored as: Never = 0, Seldom = 1, Sometimes = 2, Usually = 3 and Always = 4, **except for questions 27 & 28 in Section 6. FOR THESE QUESTIONS ONLY, reverse the scores to: Never = 4, Seldom = 3, etc.**

To calculate behavior subscale scores, use the following formulae:

"Excitability" score = Sum of scores for questionnaire items 1 + 2 ÷ 2 (i.e. items 1 + 2)/2.

"Stranger-directed aggression" score = (items 3 + 6 + 9)/3

"Owner-directed aggression" score = (items 4 + 5 + 7)/3

"Dog-directed aggression" score = (items 8 + 10)/2

"Familiar dog aggression" score = (items 11 + 12)/2

"Stranger-directed fear" score= (items 13 + 15)/2

"Nonsocial fear" score = (items 14 + 16 + 18)/3

“Dog-directed fear” score = (items 17 + 19)/2

“Touch sensitivity” score = (items 20 + 21)/2

“Separation-related problems” score = (items 22 + 23 + 24)/3

“Attachment/attention-seeking” score = (items 25 + 26)/2

“Training difficulty” score = (items 27 + 28 + 29)/3 — remember to reverse scoring order for items 27 & 28 (see above).

“Chasing” score = (items 30 + 31)/2

“Energy” score = (items 39 + 40)/2

“Miscellaneous” items other than those listed above are optional, and can be removed from the questionnaire as desired. If retained, they should be scored individually, 0–4.

NB: This shortened version of the C-BARQ was developed specifically as a behavioral screening tool for dogs relinquished to shelters by their owners. Although scores on the C-BARQ^{42} subscales are highly correlated with those on the C-BARQ^{100}, some the shorter subscales have not been formally validated, and the instrument is not therefore recommended as a research tool except under exceptional circumstances (e.g. when participants have only limited time to complete the longer survey).

© James A. Serpell,
