# Supplementary material for: Association of DNA methylation with energy and fear-related behaviors in canines
Source: Front Psychol. 2022 Dec 14;13:1025494. doi: 10.3389/fpsyg.2022.1025494 (PMC9794564; doi:10.3389/fpsyg.2022.1025494)
Supplement: Supplementary Data Sheet 4 — Supplementary file 3. [file Data_Sheet_4.pdf]

Supplemental File 3

*Targeted DNA methylation sequencing.*

The study followed the TBS-seq protocol described in Morselli et al. (2021). Specifically, they used 500 ng of extracted DNA from buccal swabs for library preparation. Fragmented DNA was End Repaired, A-tailed, and ligated to adapters. The pooled libraries were vacuum concentrated and incubated with blockers. Blockers prevent hybridization between repetitive elements and the adapter part of the libraries. These blocked libraries were hybridized and subsequently captured with magnetic beads. Then, bisulfite conversion was performed. Cutadapt was used to remove adapters from Demultiplexed Fastq files and BSBOLT to align the probes to the CanFam4 genome (Farrell et al., 2020; Martin, 2011). DNA methylation was calculated by taking a weighted average of methylation of CpGs 100 bases adjacent to the center of each probe (Rubbi et al., 2022).

*SNP annotation and genotyping*

Briefly, Rubbi et al. (2022) used the SAMtool Mpileup command to generate genotype calls from BAM files. The SAMtool command identified the aligned bases associated with each probe position. Positions were ignored if they contained cytosines or thymines and were retained if they contained adenines or guanines. They assigned genotypes (either AA, AG, or GG) according to the counts of adenine and guanine. Sites were ignored if their coverage was less than ten percent. Finally, heterozygous calls (AG) were only made for sites where the minor allele frequency was at least ten percent (Rubbi et al., 2022).
